# Redox Signaling in Chronic Kidney Disease-Associated Cachexia

**DOI:** 10.3390/antiox12040945

**Published:** 2023-04-18

**Authors:** Ana Cristina Simões e Silva, Eduardo A. Oliveira, Wai W. Cheung, Robert H. Mak

**Affiliations:** 1 Department of Pediatrics, Division of Pediatric Nephrology, Faculty of Medicine, Federal University of Minas Gerais (UFMG), Belo Horizonte 30130-100, MG, Brazil; 2 Department of Pediatrics, Rady Children’s Hospital San Diego, University of California San Diego, La Jolla, CA 92093, USA

**Keywords:** chronic kidney disease, redox signaling, cachexia, sarcopenia, muscle wasting

## Abstract

Redox signaling alterations contribute to chronic kidney disease (CKD)-associated cachexia. This review aims to summarize studies about redox pathophysiology in CKD-associated cachexia and muscle wasting and to discuss potential therapeutic approaches based on antioxidant and anti-inflammatory molecules to restore redox homeostasis. Enzymatic and non-enzymatic systems of antioxidant molecules have been studied in experimental models of kidney diseases and patients with CKD. Oxidative stress is increased by several factors present in CKD, including uremic toxins, inflammation, and metabolic and hormone alterations, leading to muscle wasting. Rehabilitative nutritional and physical exercises have shown beneficial effects for CKD-associated cachexia. Anti-inflammatory molecules have also been tested in experimental models of CKD. The importance of oxidative stress has been shown by experimental studies in which antioxidant therapies ameliorated CKD and its associated complications in the 5/6 nephrectomy model. Treatment of CKD-associated cachexia is a challenge and further studies are necessary to investigate potential therapies involving antioxidant therapy.

## 1. Introduction

Cachexia is a multifactorial syndrome that can occur in acute or chronic diseases, leading to progressive muscle wasting not completely reversed by nutritional supplementation [1]. Cachexia differs to weight loss due to insufficient caloric intake since the main alteration is muscle loss rather than fat loss. It is known that reductive/oxidative (redox) homeostasis has a role in cachexia. Cachexia is prevalent in many chronic conditions, including cancer, congestive heart failure, chronic obstructive pulmonary disease, and chronic kidney disease (CKD) [2]. These diseases lead to altered redox homeostasis and chronic systemic inflammation that, in turn, produce deleterious effects on metabolism and body composition with consequent cachexia [1,2].

Cachexia has been more extensively investigated in the process of aging [1]. Studies on age-related sarcopenia supported the importance of oxidative stress in reducing muscle mass and function. Antioxidant enzymes decrease their activity with age, thereby reducing defenses against oxidative stress [1]. It has also been verified that oxidative stress and reduced activity of antioxidant enzymes also contribute to cachexia in chronic diseases including CKD [2,3]. Oxidative stress is due to the augmentation reactive oxygen species (ROS) and reactive nitrogen species (RNS) presence inside the cells [4,5]. Superoxide anions, hydrogen peroxide, and hydroxyl radicals are ROS that are derived from aerobic metabolism. Superoxide anions are responsible for oxidative stress and are predominantly produced by nicotinamide adenine dinucleotide phosphate-oxidase (NADPH-oxidase) and by the mitochondrial electron chain. Seven different NADPH-oxidase isoforms were described, the most common being the NADPH-oxidase 4 (NOX4) in the kidney [6]. The removal of the superoxide from the body occurs by its conversion to hydrogen peroxide via the enzyme superoxide dismutase (SOD). The enzyme SOD has the isoforms SOD1, SOD2, and SOD3. There are other antioxidant systems, which can be subdivided into enzymatic and non-enzymatic categories. The enzymatic system includes SOD, catalase, glutathione peroxidase (GPx), glutathione reductase (GR), glutathione S-transferase (GST), peroxiredoxin (PRX), and thioredoxin (TRX), while the non-enzymatic system comprises ascorbic acid, alpha-tocopherol, carotenoids, flavonoids, and reduced glutathione (GSH) [7]. The importance of antioxidant systems hinges on the fact that ROS are considered toxic molecules which are able to produce damage to lipids, proteins, and DNA. Increased concentrations of ROS stimulate inflammation and cell death, while low levels are necessary for cell signaling, proliferation, and growth. Therefore, precise regulation of redox homeostasis is critical for normal cellular function. Adequate muscle cell function and metabolism depend on redox homeostasis. The abnormal elevation of ROS results in the oxidative damage of proteins, reduction of antioxidant defenses, and muscle wasting. Increased oxidative stress is also an important mechanism associated with muscle wasting during cachexia [8].

The impaired homeostasis of redox signaling also has a role in CKD-associated cachexia [2,5,7]. This narrative review aims to summarize studies about redox pathophysiology in CKD-associated cachexia and muscle wasting and to discuss potential therapeutic approaches based on antioxidant and anti-inflammatory molecules to restore redox homeostasis. The databases Pubmed and Scopus were searched using the terms “chronic kidney disease”; “chronic renal disease” and “cachexia”; “muscle wasting” and “oxidative stress”; or “redox homeostasis” and “anti-oxidant”.

## 2. Redox in Chronic Kidney Disease

Changes in redox homeostasis contribute to the progression of CKD. There is excessive production of ROS and RNS in parallel with the reduction of antioxidant mechanisms. The main consequences are hypertension (due to NO inactivation and oxidation of arachidonic acid to produce vasoconstrictive isoprostanes) [9], inflammation [due to stimulation of nuclear factor kappa B (NF-κB)] [10], fibrosis, apoptosis [11,12], and proteinuria as a result of glomerular filtration barrier impairment [13]. During inflammatory processes, ROS are produced by activated leukocytes which increase oxidative stress. Thus, a vicious cycle is established between inflammation and oxidative stress [14]. High levels of angiotensin II [15], the reduced production of NO [16], and hypertension [17] also increased ROS production in CKD.

Animal models of kidney diseases and findings in CKD patients have shown changes in redox homeostasis in CKD [7]. The studies detected high levels of ROS and RNS as well as the reduction of enzymatic and non-enzymatic systems of antioxidant molecules [7]. In the experimental model of CKD induced by 5/6 nephrectomy, the significant upregulation of NADPH-oxidase and downregulation of SOD are present in the liver and kidneys. The enhancement of superoxide is secondary to high production and reduced metabolism [9]. It was also detected that NO is inactivated by superoxide, thus resulting in high levels of systemic nitrotyrosine and a reduction of NO metabolites in urine [9]. Low amounts and low activity levels of antioxidant enzymes such as catalase, glutathione peroxidase, and the bioactive tripeptide glutathione [10] as well as decreased levels of high-density lipoprotein, apolipoprotein A-I, and thiols [11] amplified the deleterious actions of ROS. In addition, the upregulation of renal NOX4, the main isoform of NADPH oxidase responsible for superoxide synthesis in the kidney, stimulated ROS production and promoted mitochondrial damage in experimental models of polycystic kidney disease and diabetic and hypertensive nephropathies [18,19,20]. A murine model of diabetic nephropathy exhibits the downregulation of SOD1 and SOD3 in the kidney [21]. Surgically induced CKD in mutant mice with an absence of catalase production results in the fast deterioration of kidney function [22]. Besides decreased SOD and catalase concentrations, experimental models of renal diseases and CKD patients had reduced concentrations of glutathione, resulting in disease progression [23,24,25,26]. An altered antioxidant response to nuclear factor erythroid 2-related factor 2 (Nrf2) was also detected in kidney diseases. Nrf2 is a transcription factor that modulates the expression of antioxidant molecules [27]. Hyperglycemia produced oxidative stress and kidney function deterioration in Nrf2-deficient mice [28], whereas female mice with a deficiency of Nrf2 developed lupus-like autoimmune nephritis [29].

The recovery of redox homeostasis was investigated as a potential therapeutic option to delay the progression of CKD. The treatment with antioxidant molecules was evaluated in experimental models of CKD and in a few clinical trials with renal disease patients. The administration of antioxidant molecules, including melatonin [18], niacin [19], and omega-3 fatty acids [20] improved renal function and tissue damage in subtotal nephrectomized animals as a consequence of less ROS. Melatonin treatment reduced the plasma levels of malondialdehyde as well as the amount of nitrotyrosine, the infiltration of inflammatory cells, the number of interstitial cells expressing NF-κB, and the number of markers of fibrosis in kidney tissue [18]. Melatonin also attenuated creatinine elevation, proteinuria, glomerulosclerosis, and tubulointerstitial injury [18]. Niacin was administered in drinking water for 12 weeks to 5/6 nephrectomized rats and they were compared to untreated CKD animals [19]. The treatment with niacin reduced the presence of molecules related to the oxidative stress, including subunits of NOX-4, markers of inflammation, transforming growth factor (TGF)-β, and NF-κB activation. These mechanisms improved hypertension, proteinuria, and kidney tissue damage [19]. The supplementation of omega-3 fatty acid by gastric gavage for 12 weeks to 5/6 nephrectomized rats also decreased the presence of markers of oxidative stress, inflammation, and fibrosis in kidney tissue [20]. On the other hand, the administration of the SOD-mimetic tempol, despite reducing the plasma concentration of malondialdehyde and the number of superoxide-positive cells, was not able to decrease oxidative stress, inflammation, or kidney injury [21]. Another molecule with potential antioxidant and anti-inflammatory effects is curcumin, which acts as an activator of Nrf2. Accordingly, in experimental models of CKD associated with oxidative stress, the activity and expression of Nrf2 were reduced in kidney tissue and the administration of curcumin restored these parameters [30,31]. Curcumin treatment improved renal function and reduced inflammation in 5/6 nephrectomized rats [30,31]. In addition, the compound bardoxolone, a synthetic Nrf2 activator, decreased glomerulosclerosis, interstitial fibrosis, inflammation, and NF-κB activation in experimental models of kidney disease [32,33,34]. These findings supported the use of bardoxolone in clinical trials with CKD patients [35,36,37]. Unfortunately, the phase three trial (BEACON) was interrupted owing to cardiovascular events related to bardoxolone [36]. On the other hand, bardoxolone produced a sustained increase in the estimated glomerular filtration rate (eGFR) that remained until four weeks after the interruption of the administration [38]. More recently, bardoxolone has been under evaluation in patients with Alport syndrome (CARDINAL; NCT03019185), type two diabetes, CKD (TSUBAKI; NCT02316821), and in cases of autosomal dominant polycystic kidney disease (FALCON; NCT03918447). Despite the substantial amount of data on the role of ROS in CKD progression and renal diseases, the redox signaling pathways that mediated kidney injury remain to be elucidated.

## 3. Redox in Cachexia

The dynamic equilibrium between protein synthesis and breakdown determines the skeletal muscle mass. Skeletal muscle atrophy takes place when protein synthesis is slower than protein breakdown with a consequent reduction of muscle mass [39]. Following denervation, the interactions between secreted molecules and muscle cells can provoke dynamic modifications in the composition of these cells, potentially resulting in skeletal muscle atrophy [40]. Inflammation and oxidative stress are crucial causes of skeletal muscle atrophy according to earlier studies [41]. High ROS levels cause oxidative stress damage and accelerate the production of inflammatory molecules, which, in turn, potentiate muscle atrophy by increasing proteolysis and decreasing muscle synthesis and regeneration [42,43].

Increased ROS can lead to oxidative stress and changes in the skeletal muscle in chronic illnesses. An important mechanism is the stimulation of proinflammatory transcription factors, such as NF-κB. NF-κB regulates specific ubiquitin-proteasome system (UPS) genes [8] and increases the expression of the proinflammatory cytokines, IL-6 and TNF-α. These cytokines stimulate the production of ROS and the activation of UPS, resulting in a vicious cycle that worsens skeletal muscle atrophy [44,45]. The role of redox homeostasis changes has been more frequently investigated in cancer-associated cachexia [46]. The intracellular proteolytic pathways located in the skeletal muscle (proteasome, lysosomes, caspases, and calpains) are enhanced and activated in cancer-associated cachexia [47]. Muscle autophagy higher than normal limits has also been detected in cancer patients [48,49,50]. Patients with different tumors exhibited reduced activity and expression of several antioxidant enzymes associated with high superoxide levels [51].

Similar molecular changes were reported in CKD-associated cachexia. A very prevalent and harmful consequence of CKD is skeletal muscle atrophy [52]. The increased activation of proteolysis is the cause of the muscular atrophy linked to CKD [53]. Several mediators of muscle protein breakdown in CKD include the UPS, caspase-3, lysosomes, ghrelin, and myostatin. These pathways can be stimulated by CKD-related alterations including metabolic acidosis, hyperphosphatemia, inflammation, oxidative stress, and insulin resistance [43,54,55,56]. The mRNA expression for toll-like receptor-13 (TLR-13) was increased in tibialis anterior muscles from mice subjected to subtotal nephrectomy, possibly resulting in immune system overactivity. The findings of the study also indicated that TLR13 is connected to CKD-mediated insulin resistance in muscle tissue [53]. Hyperphosphatemia, a common alteration in CKD, can also contribute to muscle atrophy. Cell atrophy developed in immortalized rat L6 myotubes in a dose- and time-dependent manner when exposed to a high phosphate concentration [57]. The study concluded that high phosphate concentrations stimulated autophagy and subsequently lead to muscle cell atrophy [57]. Changes in the insulin/insulin-like growth factor-1 (IGF-1) signaling pathway also have a significant impact on the deregulation of muscle protein turnover in CKD [58].

More recently, Solagna and coworkers evaluated the cellular mechanisms behind muscle atrophy in experimental models of CKD [59]. The authors took advantage of knockout mice for the gene encoding kinesin family member 3A (Kif3a) in renal tubular epithelial cells. These animals exhibited cystic kidneys, CKD, weight loss, and muscle wasting. Several genes exhibited deregulated expression, as evidenced by the altered expression of genes involved in the respiratory chain complex, oxidative stress pathway, mitochondrial unfolded protein response, transcription factor genes, and autophagy. In two other mouse models of CKD, the authors also found increased expression of inhibin beta-A in the kidney and high circulating levels of activin A. Both molecules were also increased in the kidney tissue and in the blood of CKD patients, respectively, and were inversely correlated with the glomerular filtration rate. In addition, the blockade of activin A signaling improved muscle wasting and function in CKD animals [60]. Further research is required to investigate the potential therapeutic value of activin A in patients with CKD-associated cachexia [60]. Understanding the pathways responsible for CKD-associated cachexia will allow the development of more tailored treatments.

### The Potential Interaction of Redox with Kif3a/Inhibin Beta-A

Kif3a modulates the UPS and the autophagy-lysosome system. Numerous physiological activities, such as the cell cycle, the control of gene expression, and reactions due to oxidative stress depend on the proteasomal degradation pathway. In this regard, mice with a genetic deletion of the gene encoding Kif3a presented upregulation of the atrophy-related ubiquitin ligases Atrogin-1, Musa, Murf1, Itch, and Fbxo31 and increased expression of various autophagy-related genes, including Bnip3, Becn1, and Ambra1 in muscle tissue [60].

In addition, nine genes with pro-cachectic potential were found by microarray analysis of gene expression in kidneys from wild-type (WT) and Kif3a knockout mice. Among these genes, the inhibin beta-A (Inhba), which encodes activin A, was upregulated specifically in the kidney [60]. Myostatin, a muscle growth inhibitor, interacts with the activin A receptor type IIB and is mostly generated in skeletal muscle. The conversion of myostatin to activin A activates the downstream signaling Smad2/3 [60]. Through the forkhead box protein O (FOXO), NF-κB, and Smad2/3, oxidative stress and inflammation increased the expression of myostatin in the skeletal muscle of CKD patients [60]. All these changes contribute to muscle wasting.

## 4. Recommended Approach and Potential Treatments for CKD-Associated Cachexia

### 4.1. Non-Pharmacological Approach

Recently, rehabilitation nutrition has emerged as a strategy to overcome nutritional alterations and physical dysfunction [61]. This strategy includes the evaluation of patients’ nutritional and physical states, the definition of aims, and the simultaneous initiation of the nutritional approach and rehabilitation [62,63]. nutritional management can be founded on the International Classification of Functioning, Disability, and Health (ICF). The stimulation of a patient’s consciousness and self-management of the nutritional status have shown good results in CKD patients who need dietary support [64]. Dietary supplements should be used when the above-mentioned management fails to achieve the nutritional goals [65]. The use of tube feeding or intravenous nutrition should be considered if oral nutritional supplements are unsuccessful. Recently, the amount of protein in the diet of CKD patients was re-evaluated according to age, the etiology, and stage of CKD rather than adopting uniform protein restriction [66,67,68]. The compounds beta-hydroxy-beta-methylbutyrate (HMB), L-carnitine, and branched-chain amino acids (BCAAs) were investigated in nutritional therapy for CKD [69,70,71]. These molecules seem to improve muscle mass and function without worsening kidney function [69,70,71].

The rehabilitation for both pre-dialysis and dialysis CKD patients must include exercise therapy to improve exercise tolerance and quality of life [72]. In CKD, systematic reviews with meta-analysis found that physical exercise improved muscle mass and strength, walking ability, heart function, nutrition, and quality of life [73,74,75]. Another important measure is to stimulate the routine physical activities of CKD patients. A cohort of 20,920 patients on hemodialysis showed that the survival rates were higher in physically active individuals than in inactive ones [76]. In addition, physical activity and exercise therapy decreased the fall of the glomerular filtration rate in previously sedentary older adults [77]. On the other hand, the prescription of exercise therapy must consider the clinical conditions of the CKD patient to avoid complications. The beginning of the exercise therapy should entail low-intensity, low frequency, and short-duration activities [78]. If adequately prescribed, exercise therapy could improve physical function without decreasing the glomerular filtration rate [79].

### 4.2. Redox Signaling Associated with Nutrition and Exercise Therapy

It has been speculated that Nrf2 could be a viable therapeutic target for CKD and CKD-associated cachexia due to the overexpression of various antioxidant enzymes and cytoprotective genes that occurs when Nrf2 is activated. Different nutritional components have been evaluated as activators of Nrf2, including resveratrol, curcumin, catechins, sulforaphane (SFN), oleanolic acid, sesame oil, resistant starch, cinnamaldehyde (CA), lycopene, and selenium (Se) [80]. In diabetic rats’ kidney tissue, the administration of polydatin, a glucoside of resveratrol, increased Nrf2, heme oxygenase-1 (HO-1), SOD, and sirtuin-1 levels, thus improving the kidney tissue’s antioxidative capacity and renal dysfunction [81]. In idiopathic membranous nephropathy mice, the supplementation of resveratrol activated HO-1 expression and reduced oxidative stress via enhanced Nrf2 activity [82]. Through the Nrf2 signaling pathway, curcumin treatment in diabetic nephropathy-affected rats reduced kidney lipid accumulation and oxidative stress [83]. By increasing the expression of Nrf2/HO-1 and sirtuin-1 in rats with gentamicin-induced nephrotoxicity, curcumin supplementation decreased renal tubular cell apoptosis and oxidative stress [84]. The other nutritional components mentioned also have antioxidant properties mediated by enhancing antioxidant enzymes and/or by stimulating the Nrf2 pathway [80]. However, there is still a lack of evidence on the role of these molecules in patients with CKD since the studies are mainly performed in animal models [80].

Numerous investigations have found that following exercise, experimental animals’ Nrf2 is activated and their antioxidant enzymes are upregulated [80]. The premise to explain the modulatory role of physical exercise on oxidative stress considers that, when compared to the rest, there is a greater demand for energy during exercise, resulting in higher oxygen consumption and the production of ROS. However, oxidative stress is only temporary and stimulates the Nrf2 pathway. Then, the Nrf2 activation improves redox status and promotes muscle mass increase [80]. Unfortunately, findings in patients with CKD are very scarce and the main results were also provided by experimental studies.

### 4.3. Control of Metabolic Disturbances: Role of Vitamin D and Growth Hormone (GH)

Chronic metabolic acidosis can produce muscle wasting by enhancing glucocorticoid secretion, branched-chain ketoacid dehydrogenase, and ATP-dependent ubiquitin proteinase [85,86]. Acidemia also decreases muscle protein synthesis due to the reduction of albumin synthesis as a consequence of insulin resistance and other endocrine alterations. Other alterations associated with metabolic acidosis that contribute to cachexia are inflammation and reduction in serum leptin with consequent anorexia [87,88].

The biologically active hormonal form of vitamin D, namely 1,25-dihydroxy vitamin D_3_ [1,25(OH)_2_D_3_], which functions through the vitamin D receptor (VDR), is deficient in CKD patients [89]. VDR is expressed in muscle tissue and mediates the muscular effects of 1,25(OH)_2_D_3_ [90]. An experimental study demonstrated that 1,25(OH)_2_D_3_ deficiency contributes to muscle protein degradation mainly by stimulating UPS, which is responsible for protein degradation in several diseases including end-stage renal disease (ESKD) [91]. A subsequent experimental study of the same group found that oxidative stress in muscle tissue was also potentiated by 1,25(OH)_2_D_3_ deficiency [92]. 1,25(OH)_2_D_3_ supplementation reduced muscle protein catabolism by reversing oxidative stress and restoring antioxidant enzyme levels [92]. More recently, Zhou and colleagues [93] reported that the administration of 25(OH)D_3_, but not 1,25(OH)_2_D_3_, enhanced muscle fiber size and decreased the fat infiltration of skeletal muscle in *Ctns* knockout mice, an animal model of cystinosis. Therefore, more research is required to determine the role of the two vitamin D forms, 25(OH)D_3_ and 1,25(OH)_2_D_3_, in individuals with cachexia due to CKD.

Cachexia associated with CKD has previously been linked to growth hormone (GH) resistance [94]. For patients in hemodialysis, short-term injection of recombinant GH boosted muscle protein synthesis and muscle growth [95,96,97,98,99]. However, the beneficial mechanisms of GH in CKD-associated cachexia remain unknown. Recently, the impact of GH on cachexia linked to CKD was investigated in mice subjected to 5/6 nephrectomy [100]. The authors found that GH administration decreased uncoupling proteins (UCPs) and increased the ATP level of muscle and adipose tissue. By reducing the expression and protein level of adipose triglyceride lipase and phosphorylated hormone-sensitive lipase, GH also inhibited the breakdown of adipose tissue. Furthermore, GH reversed the molecular markers of muscle atrophy to normal levels and corrected the expression of 7 out of 12 genes linked to thermogenesis, fibrosis, and inadequate muscle and neuron regeneration in CKD mice [100].

### 4.4. Redox System Associated with Metabolic Acidosis, Vitamin D, and Growth Hormone Resistance

By activating the UPS and blocking the IGF-1/phosphatidylinositol-3 kinase (PI3K)/protein kinase B (AKT) signaling pathways, metabolic acidosis speeds up muscle atrophy [101]. While IGF-1/PI3K/AKT functions as the most significant anabolic pathway in muscle tissue, the UPS is the primary regulatory mechanism for protein breakdown in skeletal muscle [101]. Additionally, activation of AKT also inhibits protein degradation in skeletal muscle by stimulating the phosphorylation and inactivation of FOXO transcription factors. As a result, metabolic acidosis simultaneously stimulates protein breakdown and inhibits protein synthesis, as well as causing oxidative stress and inflammation in muscle tissue [101].

**Figure 1:**
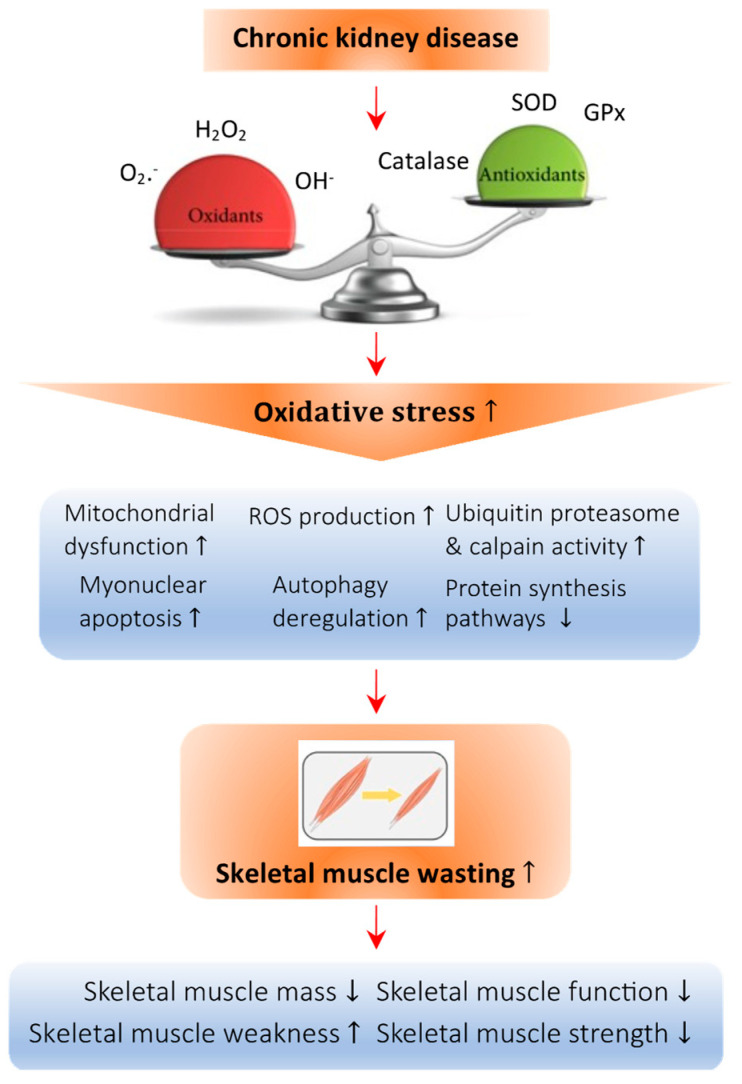
Abnormal redox signaling in CKD-associated muscle wasting. Regulation of the redox state is important in muscle cells to maintain homeostasis. Deficiency of vitamin D leads to an imbalance in the oxidative state, which increases the amount of oxidant species such as O_2_^−^, H_2_O_2_, and OH^−^ and decreases the presence of antioxidant species such as catalase, superoxide dismutase (SOD), and glutathione peroxidase (GPx). This imbalance causes oxidative damage to the cellular architecture of skeletal muscle cells and is known as oxidative stress. Specifically, oxidative stress can promote mitochondrial dysfunction and the production of ROS, raise the activity of the ubiquitin proteasome system, enhance myonuclear apoptosis and autophagy, and decrease the protein synthesis pathway, all of which lead to skeletal muscle atrophy. Arrow in the figure indicates the pathological development of the abnormal redox signaling in CKD-associated muscle wasting.

The mechanisms by which 1,25(OH)_2_D_3_ interacts with redox signaling were investigated in experimental studies [90,91,92]. The administration of 1,25(OH)_2_D_3_ inhibited the higher levels of protein oxidation in mice muscle cells induced by oxidative stress [91]. The deficiency of 1,25(OH)_2_D_3_ increased the activities of the glutathione-dependent antioxidant enzymes and reduced the activities of CAT and SOD, resulting in an altered antioxidant status. 1,25(OH)_2_D_3_ supplementation normalized all enzymatic activity [91]. SOD is known as the first line of antioxidant defense in muscle cells because it catalyzes the conversion of superoxide into oxygen and hydrogen peroxide. The treatment with 1,25(OH)_2_D_3_ increased SOD activity and was considered one of the main mechanisms for the antioxidant effect of vitamin D [90,91]. The supplementation of 1,25(OH)_2_D_3_ also corrected the nitrosative stress in mice muscle cells [91]. Figure 1 depicts the molecular mechanisms of deficiency of vitamin D implicated in CKD-associated cachexia and muscle wasting.

**Figure 2:**
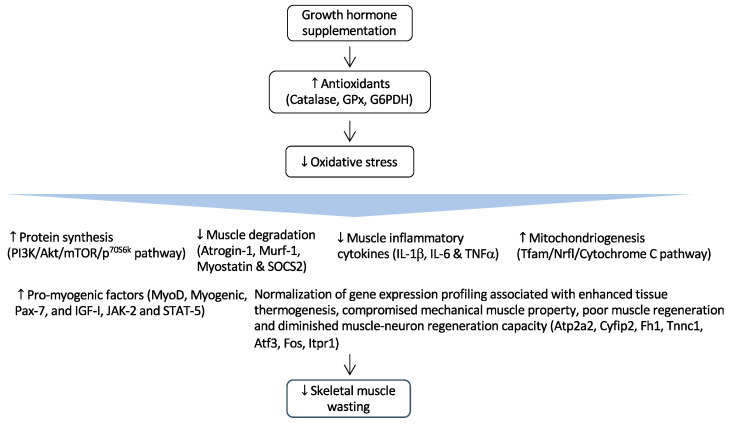
Regulation of GH and its effects in CKD-associated muscle wasting. GH supplementation activates the expression of important endogenous antioxidant enzymes such as catalase, glutathione peroxidase (GPx), and glucose-6-phosphate-dehydrogenase (G6PDH). As a result, it reduces muscle wasting by attenuating oxidative damages to critical cellular structures. Arrow in the figure indicates the beneficial effects of GH supplementation on skeletal muscle wasting.

Since increased protein catabolism and CKD-induced muscle atrophy may be caused by an aberrant GH/IGF-1 physiological axis, GH plays a key role in muscle gain [94,95]. Figure 2 illustrates the results of GH administration in CKD-associated cachexia and muscle wasting [99,101,102].

### 4.5. Potential Treatments

Anti-inflammatory medication is a potential treatment strategy for cachexia caused by CKD. Several molecules targeting inflammatory pathways were investigated in experimental models to overcome skeletal muscle atrophy. Most molecules inhibit pathways linked to oxidative stress and inflammation. In this context, molecules that blunt the NF-κB, JAK/STAT, and p38MAPK pathways prevented, at least in part, muscle atrophy. For instance, the administration of conessine, a steroidal alkaloid that antagonizes histamine, inhibited the NF-κB pathway and decreased the nuclear translocation of NF-κB [103]. The sulfur component Z-ajoene, which is present in crushed garlic, blocked the AK/STAT3 pathway and reduced p-STAT3 synthesis and nuclear translocation. These outcomes downregulated the expression of genes linked to atrophy [104]. A significant isoflavone component found in *Astragalus membranaceus*, called formononetin, protected the PI3K/AKT pathway, reduced plasma levels of inflammatory markers, slowed muscle damage, and increased protein synthesis [105]. A mouse model of cancer-related cachexia was examined using a standardized herbal combination of *Astragalus membranaceus* and *Paeonia japonica*, which is commonly used to alleviate weakness, a lack of appetite, and exhaustion. These substances reduced the inflammatory response by inhibiting the p38MAPK pathway [106]. Anakinra, a recombinant version of the natural IL-1 receptor antagonist, was administered intraperitoneally to 5/6 nephrectomized mice and restored their food intake, weight gain, fat and lean mass content, metabolic rate, and muscle function [107]. It also enhanced energy homeostasis in adipose tissue and muscle. Additionally, anakinra restored gastrocnemius weight, fiber size, and reduced muscle fat infiltration in CKD mice. It also decreased IL-6, TNF-α, and IL-1β expression in blood and muscle tissue. Anakinra decreased the expression of 17 out of the top 20 muscle genes that were differently expressed in CKD animals according to the quantitative PCR analysis. The findings support the potential role of IL-1 receptor antagonism in CKD-associated cachexia [107]. To sum up, these compounds increase muscle protein synthesis or prevent proteolysis by primarily targeting the inflammatory response and oxidative stress.

## 5. Conclusions

Although several studies support the important role of altered redox signaling in CKD and in cachexia, few of them have specifically investigated the effect on CKD-associated cachexia and almost all have been conducted in animals. Oxidative stress is increased by several factors present in CKD, including uremic toxins, inflammation, and metabolic and hormone alterations. One of the results that arises due to the interactions between oxidative stress, inflammation, and the metabolic changes is the increase in protein degradation associated with the reduction in protein synthesis, leading to loss of muscle mass among other consequences in patients with CKD. However, to date, there are few therapeutic approaches specifically aimed at controlling the abnormal redox signaling of CKD-associated cachexia. In clinical practice, some routine non-pharmacological measures for CKD, including rehabilitation nutritional (correction of acidosis and replacement of vitamin D deficiency), GH therapy, and physical exercise, may improve muscle atrophy and cachexia. These therapeutic modalities have been shown to involve antioxidant and anti-inflammatory pathways. Other potential treatments have been investigated in experimental models of CKD and mostly consisted of anti-inflammatory molecules that also reduced ROS. However, the potential role of these therapies in patients with CKD-associated cachexia has not been evaluated or is under investigation in published clinical trials. Further studies are necessary to translate findings from these basic science data to innovate novel therapy for CKD-associated cachexia.
